# Porous Carrageenan-Derived Carbons for Efficient Ciprofloxacin Removal from Water

**DOI:** 10.3390/nano8121004

**Published:** 2018-12-04

**Authors:** João Nogueira, Maria António, Sergey M. Mikhalev, Sara Fateixa, Tito Trindade, Ana L. Daniel-da-Silva

**Affiliations:** 1 CICECO-Aveiro Institute of Materials, Department of Chemistry, University of Aveiro, 3810-193 Aveiro, Portugal; jh.nogueira@ua.pt (J.N.); maantonio@ua.pt (M.A.); sarafateixa@ua.pt (S.F.); tito@ua.pt (T.T.); 2 Centre for Mechanical Technology and Automation – Nanotechnology Research Group (TEMA-NRD), Mechanical Engineering Department, Aveiro Institute of Nanotechnology (AIN), University of Aveiro, 3810-193 Aveiro, Portugal; mikhalev@ua.pt

**Keywords:** carrageenan, hydrochar, activated carbon, adsorption, ciprofloxacin

## Abstract

Porous carbon materials derived from biopolymers are attractive sorbents for the removal of emerging pollutants from water, due to their high specific surface area, high porosity, tunable surface chemistry, and reasonable cost. However, carrageenan biopolymers were scarcely investigated as a carbon source to prepare porous carbon materials. Herein, hydrochars (HCs) and porous activated carbons (ACs) derived from natural occurring polysaccharides with variable sulfate content (κ-, ι- and λ-carrageenan) were prepared and investigated in the uptake of ciprofloxacin, which is an antibiotic detected in water sources and that poses serious hazards to public health. The materials were prepared using hydrothermal carbonization and subsequent chemical activation with KOH to increase the available surface area. The activated carbons were markedly microporous, presenting high specific surface area, up to 2800 m^2^/g. Activated carbons derived from κ- and λ-carrageenan showed high adsorption capacity (422 and 459 mg/g, respectively) for ciprofloxacin and fast adsorption kinetics, reaching the sorption equilibrium in approximately 5 min. These features place the ACs investigated here among the best systems reported in the literature for the removal of ciprofloxacin from water.

## 1. Introduction

**Scheme 1:**
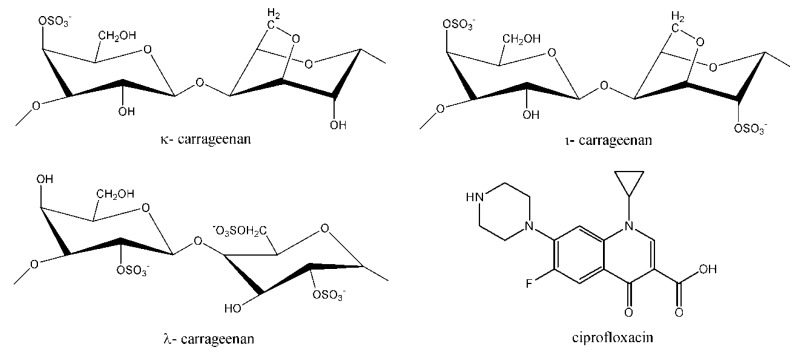
Structural formulas of the disaccharide units of κ-, ι- and λ-carrageenan, and ciprofloxacin.

Emerging pollutants are a vast series of man-made chemicals such as cosmetics, pesticides and pharmaceuticals that are essential to modern society and whose production has increased dramatically over the last century. The continuous and uncontrolled discharge of such substances into the environment, from distinct pollution sources, contributes to their accumulation in the aquatic compartments, with potentially harmful effects [1,2]. Effects on human health, environment and aquatic ecosystems are still poorly studied, but may include endocrinal disruption, promotion of antibiotic resistance, and chronic toxicity [3,4]. Unfortunately, wastewater treatment plants (WWTP) are currently unable to completely remove most of these compounds [5,6]. This is the case of ciprofloxacin (CIP, Scheme 1), an antibiotic that belongs to the class of quinolones and is used to treat several bacterial infections in animals and humans [7]. Ciprofloxacin can be found in wastewater, due to improper disposal and incomplete metabolization of the drug in humans. The average CIP removal rate in WWTPs is about 60% [5], resulting in wastewater effluents with concentrations that can still exceed 1 µg/L [8]. Furthermore, the detected amount of CIP in wastewaters discharged from hospitals and drug production units is much higher, up to 150 μg/L and 30 mg/L, respectively, which is potentially harmful to human health and ecosystems [9,10,11]. A recent study has indicated ciprofloxacin as the most widespread antibiotic in sea water in the Antarctic [12]. The presence of CIP in water sources poses serious hazards to public health also because it can promote antimicrobial resistance in certain pathogenic microorganisms [12,13,14].

Given the limitations of conventional treatments implemented in WWTPs, new, sustainable and effective technologies are sorely needed. Several methods have been proposed for the removal and degradation of CIP from water, including advanced oxidation processes [15], photocatalytic treatment [16], electrocoagulation [9], biodegradation [17] and adsorption [18,19]. Among these methods, adsorption is an interesting process in view of its simplicity of implementation, low cost, high efficiency, and less production of toxic intermediates. In order to achieve high performance adsorptive separation, it is crucial to select sorbent materials with high capacity, chemical selectivity and fast rate of adsorption. This quest has boosted the development of new types of efficient sorbent materials. A variety of carbon-based materials have been investigated as sorbents for the uptake of CIP [20,21,22,23]. Among these materials, porous carbon such as activated carbons (ACs) have received remarkable attention [18,24,25,26], owing to high specific surface area, high pore volume, micro- or mesoporosity, tunable surface chemistry, and reasonable cost. These features are crucial to achieve high performance in sorbent materials at low cost. In this context, it is important to consider that the textural properties of ACs are tightly related to the raw materials used as precursors and the method of preparation [27]. Activated carbon production usually comprises two-steps [27]. First, the precursor is carbonized through pyrolysis at high temperatures or hydrothermal carbonization at mild temperatures. In the second step, the hydro- or pyrochars are submitted to an activation process, with the aim of increasing the surface area. In order to reduce the production cost, porous carbon materials and activated carbons are commonly obtained using raw biomass and carbohydrates as precursors [27]. Natural precursors rich in structural heteroatoms (e.g., chitosan) have been less investigated but are particularly attractive as they offer the chance of producing hetero-doped carbon structures with additional functionalities of interest for several applications [28,29].

Carrageenan is a family of linear sulfated polysaccharides composed of galactose and anhydrogalactose units, extracted from red seaweeds [30]. The most commonly used are κ-, ι- and λ-carrageenan, that comprise one, two or three sulfated groups per disaccharide unit, respectively (Scheme 1). Carrageenans have been widely used as emulsifying or gelling agents in the pharmaceutical and food industries [30,31]. Owing to its natural abundance and chemical functionality, carrageenan have attracted attention for sorption applications, namely in the uptake of pesticides and pharmaceutical contaminants from water [32,33,34,35]. However, to the best of our knowledge, carrageenan has been scarcely investigated as a carbon source to prepare porous carbon materials. Furthermore, studies on carrageenan-derived porous carbons were limited to energy-related applications [36,37]. For example, Fan and co-workers [37] reported that mesoporous carbon microspheres prepared from carrageenan (undefined type) present good electrochemical capacitive performance owing to high surface area, narrow pore size distribution and optimized micro- and mesoporous structure.

In this work, we have investigated the use of three distinct carrageenans with variable sulfate content (κ-, ι- and λ-carrageenan) as precursors submitted to hydrothermal carbonization in the preparation of activated carbons and subsequent chemical activation with potassium hydroxide (KOH). Furthermore, we have explored the application of the prepared materials as sorbents for the removal of the antibiotic ciprofloxacin from aqueous solutions.

## 2. Materials and Methods

### 2.1. Chemicals

Three types of carrageenan (κ, ι and λ-carrageenan) were acquired from Honeywell Fluka (Seelze, Germany). Potassium hydroxide (>86%) was purchased from Labchem (Zelienople, PA, USA) and hydrochloride acid (HCl, 37% *v*/*v*) was purchased from VWR International (Radnor, PA, USA). Ciprofloxacin hydrochloride (99%) was purchased from Sigma-Aldrich (St. Louis, MO, USA). Milli-Q water was obtained through Synergy equipment (Millipore, 0.22 µm filter) and absolute ethanol (CH_3_CH_2_OH) was purchased from Honeywell Fluka (Seelze, Germany).

### 2.2. Preparation of the Activated Carbons

Synthesis of the activated carbons involved two main steps, a hydrothermal treatment of the carrageenan, followed by chemical activation [37]. In a typical procedure, 0.87 g of carrageenan (κ, ι or λ-carrageenan) were dissolved in water (17.5 mL) under stirring, placed in a Teflon autoclave and subjected to 200 °C for 20 h. The resulting hydrochar, a dark precipitate, was collected by centrifugation (15 min, 6000 rpm, Hettich Zentrifugen, EBA 20), washed with water and ethanol several times, and freeze-dried (Coolsafe Touch, Labogene). The hydrochars were identified according to their carrageenan precursor–HC-κ, HC-ι and HC-λ. These materials were chemically activated by KOH in a 1:4 HC:KOH weight ratio (physical mixture) in a tube furnace, under nitrogen atmosphere, at 700 °C (4 °C/min) for 4 h. The obtained materials were washed with hydrochloric acid (2 M), followed by water to remove all potassium, and then suspended in ethanol and dried at 60 °C overnight. The resulting activated carbons were identified as AC-κ, AC-ι and AC-λ according to their AC precursor.

### 2.3. Adsorption Experiments

The ability of the activated carbons to uptake ciprofloxacin (CIP) from water was assessed through batch adsorption experiments in polypropylene containers. AC samples were precisely weighted and added to a CIP aqueous solution of known concentration in deionized water and were continuously shaken using a vertical rotator at a constant rotation speed (30 rpm) under isothermal conditions (25 ± 1 °C). The starting point of the uptake experiment was coincident with the beginning of the stirring process. A comparison between the adsorption performances of the HC and AC materials was preliminary assessed by using 0.5 mg/mL of carbon at pH 5. CIP solutions were prepared daily by diluting the corresponding stock solution. In each experiment, aliquots were collected for analysis at different times. The carbon materials were separated from the medium by centrifugation (13,300 rpm, 5 min, Spectrafuge 24D, Labnet). The concentration of CIP in the supernatant was determined by measuring the absorbance at 273 nm using a UV–Vis spectrophotometer (Cintra 303, GBC). The calibration curve was built with CIP standards with concentrations between 0.12 and 12 mg/L.

The amount of adsorbed CIP at time *t* (*q_t_* in mg/g) was estimated from the mass balance between the initial CIP concentration (*C_0_* in mg/L) and concentration at time *t* (*C_t_* in mg/L) in the solution, as displayed by Equation (1), where *V* is the total volume of CIP solution (L) and *m* is the mass of the dry weight of the sorbent.

(1) $$q_{t}=\frac{\left(C_{0}-C_{t}\right)V}{m}$$

The removal percentage (R) of CIP was calculated using Equation (2):

(2) $$R=\frac{C_{0}-C_{t}}{C_{0}}\;\times 100$$

Control uptake experiments, i.e., in the absence of carbon materials, were also carried out in parallel under the same conditions to inquire about CIP losses due to adverse effects.

#### 2.3.1. Effect of pH, Sorbent Dosage and Equilibrium Isotherms

The effect of pH on adsorption was firstly investigated. The pH of CIP solutions was adjusted using ammonia (25%) or hydrochloric acid (37%). Then the adsorption capacity at equilibrium (*q_e_*) was determined at pH 6 and 24 h contact time for an initial CIP concentration of 50 mg/L with variable sorbent dosage (0.05, 0.1, 0.25, 0.4 and 0.5 mg/mL of AC). The amount of adsorbed CIP at equilibrium (*q_e_*, mg/g) was assessed by UV-Vis spectroscopy and calculated using Equation (1) for *C_t_* = *C_e_*, where *C_e_* (mg/L) is the concentration of CIP at equilibrium. The isotherm curves were built by plotting *q_e_* against *C_e_* (mg/L).

#### 2.3.2. Effect of Contact Time

To investigate the kinetics of adsorption, the time profile of CIP adsorption was assessed. Typically, 10 mg of activated carbon (accurately weighted) were added to 20 mL of CIP solution (50 mg/L, pH 6). With the mixture being shaken, aliquots of 0.7 mL were collected over time, at 25.0 ± 1.0 °C. The amount of CIP adsorbed onto the AC samples at each time interval (*q_t_*, mg/g) was determined using Equation (1) and plotted against time (*t*, min).

### 2.4. Materials Characterization

Nitrogen physisorption experiments were performed with a Gemini V2.0 Micromeritics Instrument (Micromeritics, Norcross, GA, USA) to investigate the textural properties of the carbonaceous materials. The specific surface area of the materials was determined from N_2_ adsorption/desorption isotherms, using the Brunauer–Emmett–Teller (BET) equation [38]. The total pore volume (*V_T_*) was defined as the volume of liquid nitrogen corresponding to the amount adsorbed at a relative pressure *p/p_0_* = 0.99 (Gurvitch rule) [39]. The micropore volume, *V_micro_*, was calculated according to the Dubinin–Radushkevitch method [40]. The average pore size (*d_P_*) was calculated using the ratio *4V_T_/S_BET_* that considers pores with cylindrical shape. Elemental analysis of carbon, hydrogen and sulphur was performed on a Leco Truspec-Micro CHNS 630-200-200 (LECO, Saint Joseph, MI, USA). Fourier transform infrared (FTIR) spectra of the materials were obtained using a Bruker Optics Tensor 27 spectrometer (Bruker, Billerica, MA, USA) coupled to a horizontal attenuated total reflectance (ATR) cell, using 256 scans at 4 cm^−1^ resolution. Raman spectra were acquired in a combined Raman-AFM confocal microscope WITec alpha300 RAS+ (WITec, Ulm, Germany). An Nd:YAG laser operating at 532 nm was used as excitation source with the power set to 1 mW. A 100× objective was used to view samples with an integration time of 2 s for each spectrum and 10 acquisitions. The intensity values of the Raman bands of the carbon materials for the ratio I_D_/I_G_ calculation were obtained by fitting the Gaussian function in Project five^+^ software (WITec, Ulm, Germany). The carbon materials were analysed by powder X-ray diffraction (XRD) in a Rigaku Geigerflex Dmax-C diffractometer (Rigaku, Tokyo, Japan) equipped with a CuKα monochromatic radiation source with 0.026° as step size and 350 s as time per step. The morphology and size of the particles was analysed by scanning electron microscopy (SEM) using a Hitachi Su-70 microscope (Hitachi, Tokyo, Japan) operating at 15 kV. Samples for electron microscopy analysis were prepared by evaporating particle suspensions (in ethanol) on a cooper grid coated with an amorphous carbon film. Zeta potential measurements were performed in aqueous solutions of the particles to assess the surface charge of the particles, using Zetasizer Nano ZS equipment from Malvern Instruments (Malvern, United Kingdom).

## 3. Results and Discussion

### 3.1. Characterization of the Carbon Materials

**Figure 1:**
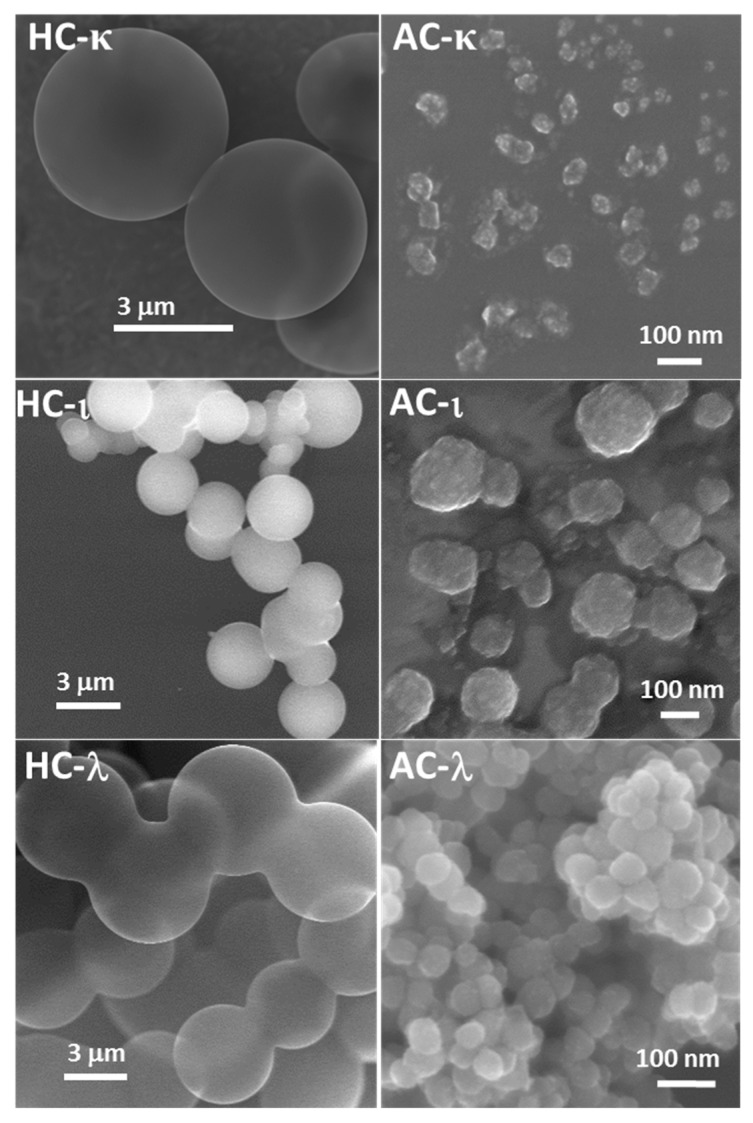
Electron micrographs of carrageenan-derived hydrochars (**left**) and activated carbons (**right**).

The electron microscopy analysis of the HCs and ACs prepared using different carrageenan types as precursors unveiled distinct morphological and microstructural features (Figure 1). The hydrothermal carbonization of carrageenan produced spheroidal carbon particles with a smooth surface and an average diameter between 3 and 5 µm, respectively (Figure 1 and Table 1). Hydrothermal carbonization of λ-carrageenan formed bottleneck junctions between adjacent HC-λ particles. Upon activation with KOH the particle size decreased drastically to nanometric dimensions, with the average size ranging between 50 and 150 nm, and the particles exhibited a rough surface. The spherical shape of the hydrochars HC-ι and HC-λ was preserved after KOH activation, in agreement with previous findings reported for spherical-activated carbons prepared from carrageenan (undefined type) using identical KOH activation conditions [37]. In contrast, the activated carbons prepared from κ-carrageenan presented irregular shape.

Among the hydrochars, HC-λ presented the highest oxygen content as determined by elemental microanalysis. Although carrageenans are sulphonated polysaccharides, sulfur was detected in a vestigial amount only for ι-carrageenan-derived hydrochars (0.4 wt%) (Table 1). As expected on the basis of previous studies, activation with KOH resulted in an increase of carbon content and a decrease of oxygen content in the materials prepared from κ- and λ- carrageenan [41]. Unexpectedly, the activation barely affected the elemental composition of ι-carrageenan-derived AC, whose carbon content was lower than in the other activated carbons, but has higher oxygen content. 

**Figure 2:**
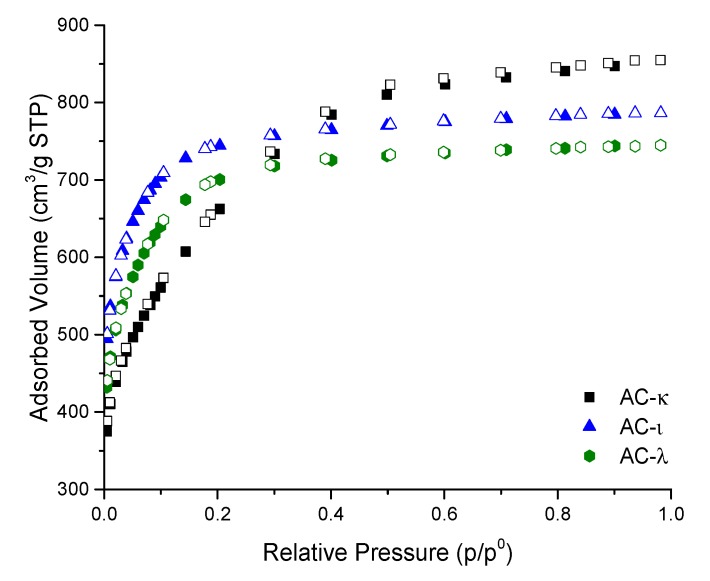
N_2_ adsorption (full symbols) and desorption (empty symbols) isotherms for activated carbons at 77K.

The textural properties of the hydrochars and the activated carbons were analyzed by N_2_ sorption/desorption technique. The results are included in Table 2. The hydrochars have a specific surface area (*S_BET_*) between 4.9 and 30 m^2^/g and low total porosity (*V_T_*), which are comparable with the values reported for hydrochars prepared from polysaccharides [37]. The *S_BET_* was higher for HC-ι spheres, which is in line with the smaller average particle size observed in SEM images, combined with the higher value of total porosity. The surface area markedly increased through KOH activation in agreement with the decrease of particle size and increase of surface roughness observed by electron microscopy analysis. Furthermore, the activation process makes the carbons highly porous, as expected on the basis of previous works [42,43]. Noteworthy, all ACs present a specific surface area above 2300 m^2^/g, and up to 2800 m^2^/g for the AC derived from ι-carrageenan, which are values superior to the specific surface area of commercial activated carbons [44]. The N_2_ adsorption-desorption isotherms (77 K) are type I (Figure 2), which is characteristic of materials with marked microporous character [45]. The volume of micropores (*V_micro_*) calculated using the Dubinin–Radushkevitch equation was high for all AC samples, from 0.8 to 1.1 cm^3^/g, and is in line with the microporosity features demonstrated in the isotherm profile. 

**Figure 3:**
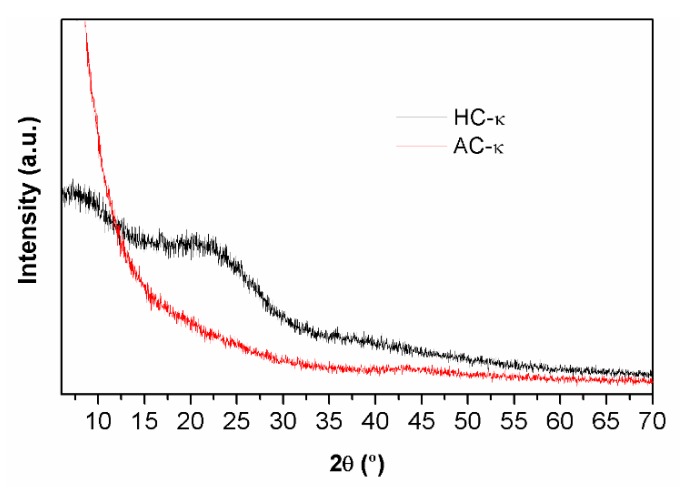
Powder XRD pattern of κ-carrageenan-derived hydrochar (HC) and activated (AC) carbons.

The powder XRD of the hydrochars shows a single broad feature centered at 23° corresponding to (002) of graphite, which suggests a material composed of amorphous carbon with low graphitization (Figure 3 and Figure S1, Supporting Information) [37]. The absence of marked reflection peaks in the XRD patterns of activated carbons indicates that the level of structure order decreases after activation.

**Figure 4:**
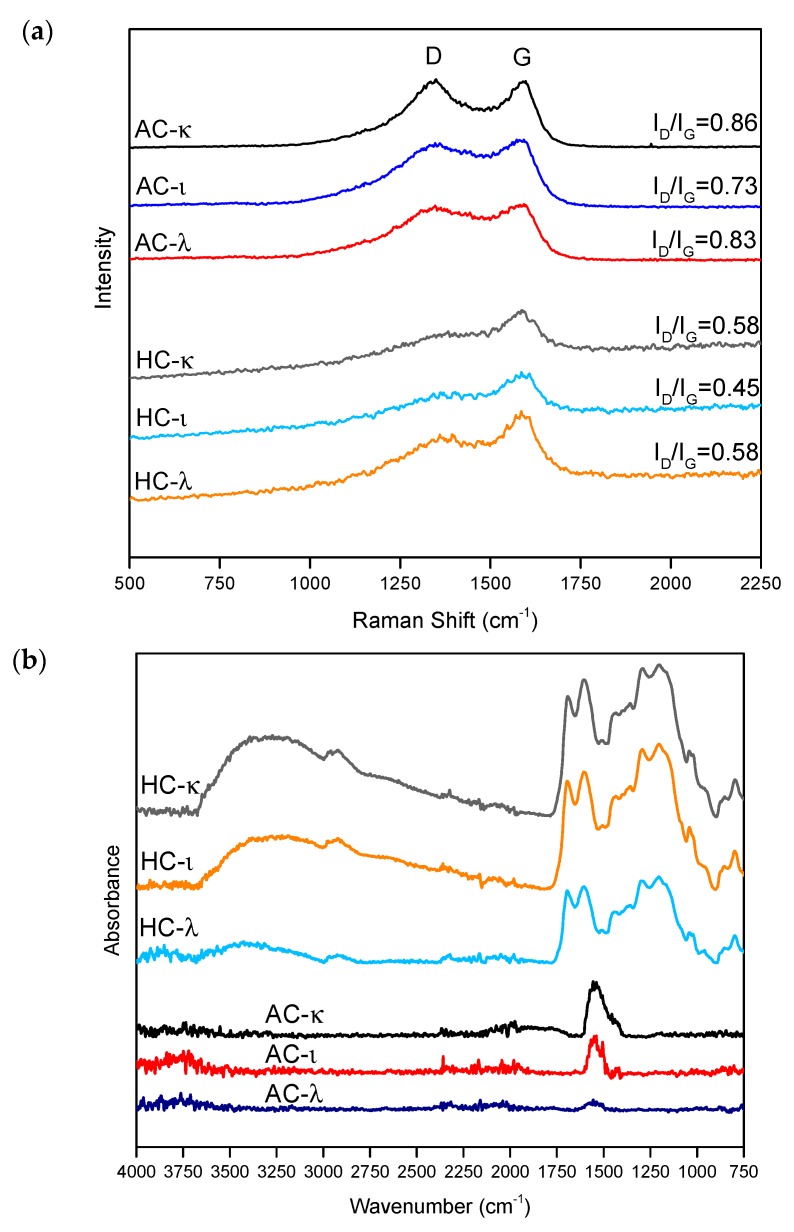
Raman (**a**) and ATR-FTIR (**b**) spectra of hydrochars (HC) and activated (AC) carbons.

Figure 4a depicts the Raman spectra of the materials before and after activation with KOH. The spectra show two bands at around 1360 cm^−1^ (D band) and 1586 cm^−1^ (G band) which are typical of carbon materials. The G band is attributed to the C-C bond in ordered graphitic structures and indicates the presence of sp^2^-hybridized C atoms present in materials such as graphene, carbon nanotubes and amorphous carbon [46]. The D band denotes the presence of defects and disorder in the sp^2^ structure, namely due to the presence of sp^3^-hybridized carbon atoms. The intensity ratio between the D-band and G-band (*I_D_/I_G_*) is an important parameter to monitor the disorder in the carbon structure of the materials. The *I_D_/I_G_* ratio was identical in the hydrothermal carbons prepared from κ- and λ-carrageenan and slightly lower when ι-carrageenan was used. Overall, the *I_D_/I_G_* value increased after activation, which indicates that the degree of graphitization decreased after KOH activation. This is in line with previous works that reported that activation with KOH at high temperatures (700–900 °C) and high KOH/C ratios (4:1 and 3:1, *w*/*w*) favors the conversion of the conjugated aromatic structure into sp^3^-hybridized C atoms [37,47].

The chemical identity of the carbon materials before and after activation was assessed by ATR-FTIR spectroscopy (Figure 4b) and the most relevant bands were assigned as depicted in Table S1 (Supporting Information). All hydrochars exhibited a broad band at 3000–3600 cm^−1^ that is ascribed to O-H stretching vibrations from surface hydroxyl groups and adsorbed water molecules. The bands at 2929 and 2879 cm^−1^ confirm the presence of aliphatic carbons, –CH_2_- and –CH_3_ [48]. The overlapping bands with peaks at 1695 cm^−1^ and 1606 cm^−1^ are due to carbonyl (C=O) stretching vibrations and the stretching of C=C in aromatic rings, respectively [49]. The broad band at 1298 cm^−1^ is ascribed to C-O bending vibrations [50]. The band at 798 cm^−1^ is attributed to the C-H vibration in aromatic structures [49]. After activation, the bands due to OH, C=O and C-O vibrations are less intense (or not observed), which indicates the decrease of surface functionalization with oxygen-containing groups. The band at 798 cm^−1^ disappears, which indicates the decrease of aromatization after activation and is in agreement with the Raman observations presented above. Still, vibrations of aromatic C=C bonds can be detected through the most intense band in the region 1520–1560 cm^−1^ [48]. 

**Figure 5:**
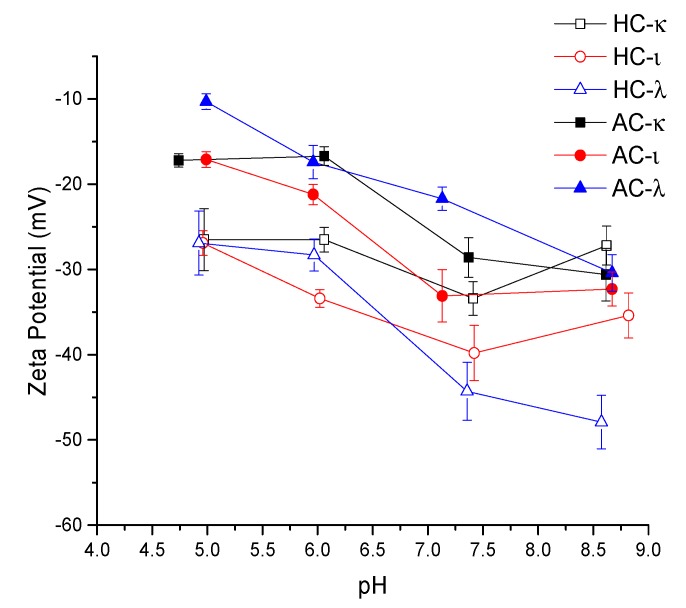
Zeta potential titration for hydrochars and activated carbons.

Determination of zeta potential (ζ) was performed to assess the surface charge of the carbon materials. The measurements were performed at pH ranging from approximately 5 to 9, which includes the normal range for pH in surface water systems (6.5–8.5). Zeta potential measurements revealed that all the carbon materials display a negative surface within the pH range tested (Figure 5). Negative ζ-values in the hydrochars are due to ionized oxygen containing groups present at the surface of the carbons. Overall, the ζ-values of activated carbons range from ca. −10 mV at pH 5 to −30 mV at pH 8.5 and are less negative than in hydrochars, which is in line with elemental microanalysis and FTIR results that denote a decrease of oxygen-containing groups after activation. However, highly negative surface charge of activated carbons at pH values above 7 suggests high colloidal stability of these materials and strong stability against aggregation that could favor the adsorptive properties.

### 3.2. Uptake of Ciprofloxacin From Water

**Figure 6:**
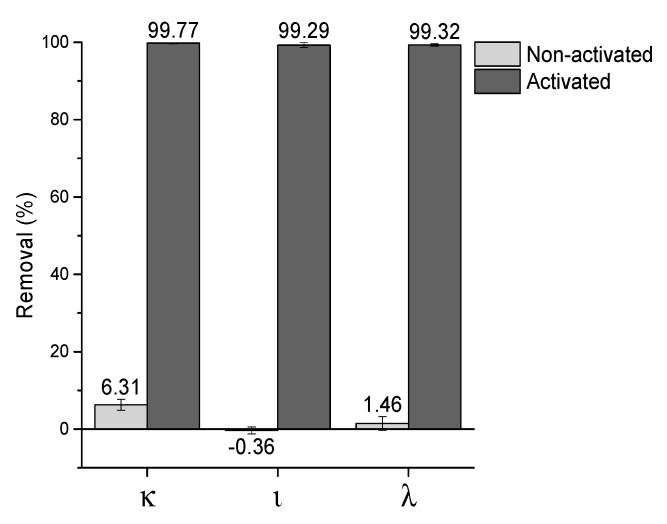
The performance of the hydrochars and activated carbons for the removal of ciprofloxacin from water (conditions: 50 mg/L CIP, pH = 5, 24 h contact time, sorbent dosage: 0.5 mg/mL).

The performance of the carbonaceous materials before and after KOH activation in the uptake of ciprofloxacin was investigated for the same initial concentration of CIP (50 mg/mL, deionized water) at pH 5, with 24 h contact time. The results clearly show that activated carbons have a higher capacity to adsorb CIP than the hydrochars (Figure 6). Overall, the CIP removal by hydrochars is very low (<10%) but increases to values above 99% when activated carbons are used in similar operational conditions.

#### 3.2.1. Effect of pH on Adsorption in Aqueous Medium

**Figure 7:**
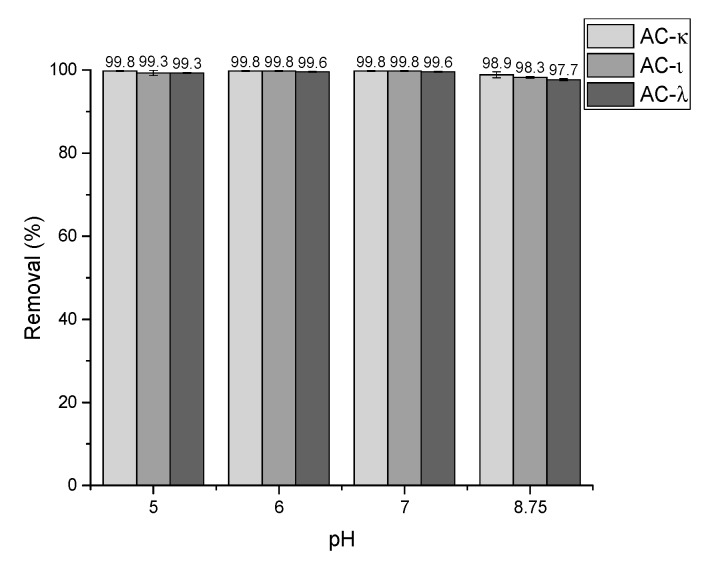
Removal of ciprofloxacin (50 mg/L) from water samples at distinct pH using the activated carbons (conditions: 24 h contact time, sorbent dosage: 0.5 mg/mL).

The adsorptive performance of the activated carbons was investigated in the pH range 5–9 for 24 h contact time for an ciprofloxacin (CIP) concentration of 50 mg/L (Figure 7). Control experiments were carried out in parallel without sorbents and under similar conditions of pH and contact time, and have shown negligible losses of CIP (<2%, data not shown). Therefore, the decrease of CIP concentration in the presence of the activated carbons was ascribed to adsorption phenomena. The CIP removal was above 99% for pH values between 5 and 7, and slightly decreased to 98–99% at pH = 8.8. According to the zeta potential results (Figure 5), all the ACs present a negatively charged surface for pH 5–9. However, CIP is present in the form of distinct ionic species within this pH range (Figure S2, Supporting Information) [51,52]. At pH = 5, CIP is mainly in the cationic form due to protonation of the aminic groups but at pH = 7 it is mostly as zwitterions. Both forms contain protonated amine groups that could interact electrostatically with the negatively charged surface of the carbons. Still, at pH = 8.8 about half of the CIP molecules are in the anionic form and thus are less prone to interact with the sorbent’s surface via electrostatic interactions. This could explain the decrease on the CIP adsorption at pH = 8.8 and suggests that cation-π interactions between CIP molecules and π-electrons of the carbon structure play a role in the sorption mechanism [53]. Yet, even at pH = 8.8 the removal capacity was high. This indicates that the sorption mechanism may involve other pathways such as π-π interactions, hydrogen bonding and hydrophobic interactions [54]. The kinetics and equilibrium studies were performed at pH 6, which is within the normal range of pH in surface water systems.

#### 3.2.2. Effect of Adsorbent Dosage

**Figure 8:**
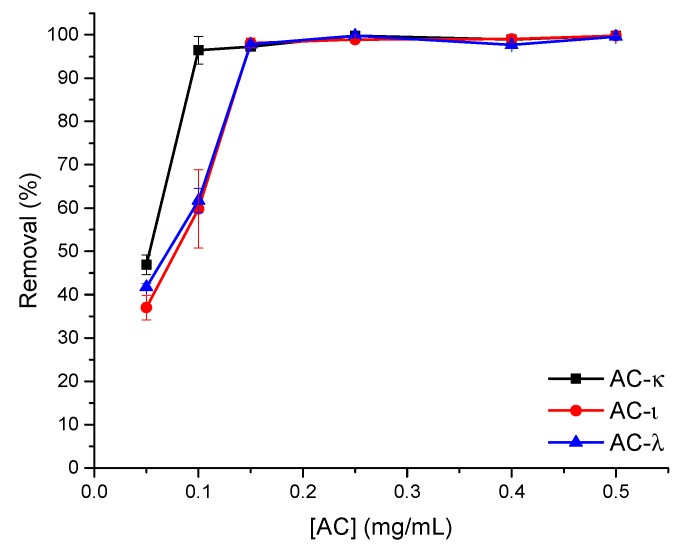
Effect of the adsorbent dosage in the removal of CIP from water, using the carrageenan-based activated carbons (conditions: 50 mg/L CIP, pH = 6, 24 h contact time).

The effect of the adsorbent dose on the adsorption of ciprofloxacin was investigated in the range 0.05 to 0.5 mg/mL. The results (Figure 8) show that CIP removal increases with the increase of the amount of sorbent until it reaches a plateau. At 0.25 mg/mL or higher dosages, all the carbons display a good performance (≥98–99%) in the removal of ciprofloxacin. At a dosage of 0.1 mg/mL, the removal of CIP is markedly higher using the carbon AC-κ (>95%) than with AC-λ (61.7%) or AC-ι (59.9%), which suggests a better adsorption performance for the activated carbon prepared from κ-carrageenan.

#### 3.2.3. Isotherm Studies

**Figure 9:**
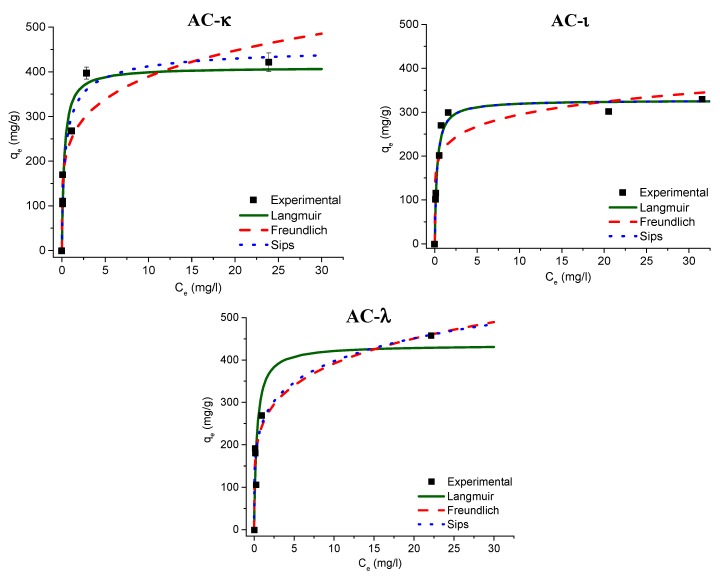
Isotherm data and the corresponding model fitting for the adsorption of ciprofloxacin on the activated carbons.

The equilibrium adsorption capacity of CIP (q_e_) as a function of equilibrium concentration of CIP (*C_e_*) is depicted in Figure 9. The equilibrium data were analyzed using several common isotherm models: the Langmuir [55] and Freundlich [56] isotherms, which are two-parameter isotherms, and the Sips isotherm [57,58], also named the Langmuir-Freundlich method, which is a three-parameter isotherm (See Table S2, Supporting Information for model equations). The isotherm equations were fitted to experimental data by nonlinear regression analysis. The goodness of the fitting was assessed based on the analysis of the correlation coefficient (*R^2^*) and Chi-square test value (χ^2^) (Equations (S1) and (S2), Supporting Information). The goodness of the fittings and model parameters are shown in Table 3.

Overall, the Sips model provides the best correlation with the experimental data, with *R^2^* ranging from 0.9315 to 0.9808. The Sips (or Langmuir-Freundlich) isotherm is, as the name indicates, a combination of Langmuir and Freundlich isotherms. At low sorbate concentration, the Sips equation reduces to a Freundlich isotherm, while at high sorbate concentrations, it predicts the sorption capacity of a monolayer, characteristic of the Langmuir isotherm. [59]. This isotherm is capable of modeling both homogeneous and heterogeneous binding surfaces. The exponent *m* is the heterogeneous index that varies from 1, in a homogeneous surface, to *m* < 1, in a heterogeneous surface. For AC-ι, *m* = 1 indicates that the surface of this sorbent is homogeneous, i.e., all binding sites are energetically equivalent. In this case, the Sips isotherm is reduced to Langmuir isotherm and the parameter *a* corresponds directly to the Langmuir isotherm constant K_L_ (Table 3). Whereas the value of the exponent *m* on Sips equation was <1 for both AC-κ and AC-λ, which indicates heterogeneous surfaces in these sorbents. For AC-λ, both *a* and *m* approaches 0 and the Sips equation tends to reduce to Freundlich isotherm. Indeed, for this AC the goodness of fit by Sips and Freundlich models was identical. The Freundlich isotherm is an empirical model with wide application in heterogeneous systems, which assumes that the adsorption could take place via multiple layers instead of a single layer [56]. 

A direct comparison between the ACs shows that the maximum adsorption capacity of AC-ι (330 mg/g) is lower than for AC-κ (422 mg/g) or AC-λ (459 mg/g). Among the carbons prepared, the sample AC-ι presented the highest specific surface area (2805 m^2^/g) and the lowest carbon content (72 wt%), which might indicate that the carbon content on the surface might have a relevant role on the adsorption of ciprofloxacin by these materials.

#### 3.2.4. Effect of Contact Time and Kinetic Studies

**Figure 10:**
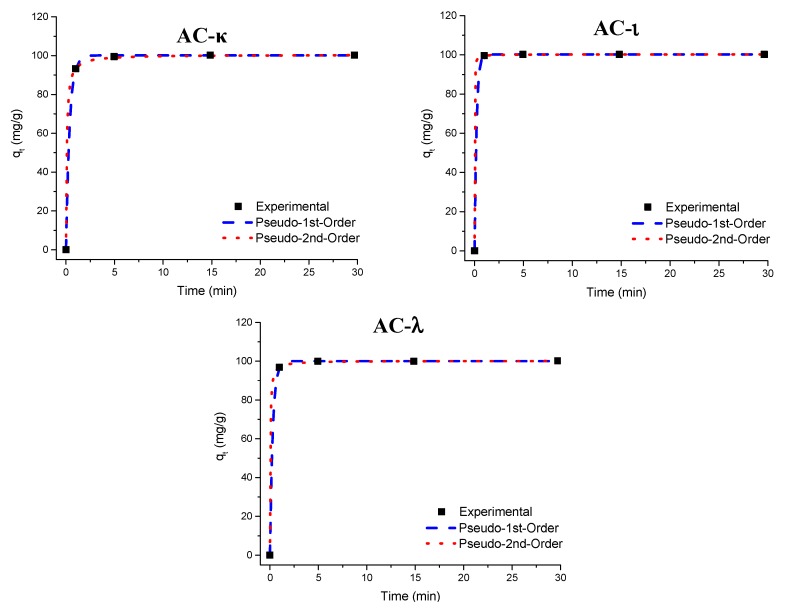
Time profile of CIP adsorption capacity over 30 min and the corresponding kinetic model fitting using pseudo 1st and pseudo 2nd order equations.

The time profile of CIP adsorbed onto the ACs was assessed for an initial concentration of 50 mg/L, in order to investigate the kinetics of sorption. It was found that 5 min of contact time was sufficient to achieve the optimal performance, with CIP removal higher than 99% (Figure 10). Overall, the adsorption kinetics with these ACs is much faster than others ACs tested in the uptake of CIP in similar conditions and reported in the literature. For example, bamboo-derived ACs tested in identical dose (0.5 mg_AC_/mL) and similar initial CIP concentration (40 mg/L) required several hours to achieve the sorption equilibrium and maximum sorption performance [24]. 

It is well accepted that the solid–liquid adsorption step involves several steps [60,61]. Initially the sorbate species migrate from the bulk solution to the solid/liquid interface (bulk diffusion). Then the sorbate diffuses across the liquid film surrounding the solid to the surface of the sorbent (film diffusion). Afterwards, the sorbate diffuses in the liquid within the pores (intra-particle diffusion). Finally, the sorbate reacts with the active sites of the sorbents surface through chemical reaction or physical adsorption. The use of kinetic models allows elucidating the adsorption mechanism. The kinetic adsorption data was fitted to two kinetic equations commonly used: the pseudo-first order equation [62] and the pseudo-second order equation (see Supporting Information for model equation model-Table S3) [63]. These models assume that the interaction of the sorbate with the active sites is the rate-limiting step and is of chemical nature [61]. The kinetic parameters and goodness of the fits, obtained by non-linear regression, are reported in Table 4 and the kinetic fittings are shown in Figure 10 and Figure S3 (Supporting Information). Both equations provided a very good fit to the experimental data, with a high coefficient of determination (*R^2^* > 0.9999) and low chi-square values (χ^2^). These results indicate that the chemisorption of CIP molecules onto ACs surface is the rate limiting mechanism of CIP adsorption.

### 3.3. Comparison with Other Sorbents

The measured maximum adsorption capacity was 422 mg/g, 330 mg/g and 459 mg/g for AC-κ, AC-ι and AC-λ activated carbons, respectively. Table 5 shows the CIP adsorption capacity reported for other carbonaceous sorbents, and AC-κ and AC-λ can be regarded as some of the most effective adsorbents for CIP. The fast adsorption kinetics of these ACs when compared to other materials with high sorption capacity (H_3_PO_4_ chemically-activated carbon from bamboo–AC_bamboo_/H_3_PO_4_) is also an advantage. The combination of fast CIP uptake and high sorption capacity makes these ACs very attractive for the efficient uptake of CIP from water.

As for pilot studies, a study worth mentioning has investigated the efficacy of a fluidized activated carbon pilot as tertiary treatment to remove a wide range of pollutants from wastewater treatment plant effluents. While many pollutants are shown to be removed, there was an average 87% removal for ciprofloxacin [66], which is a favorable indicator of the potential of the ACs developed herein for CIP removal in continuous flow conditions. 

## 4. Conclusions

Hydrochars and porous-activated carbons prepared from three distinct carrageenan polysaccharides, κ-, ι- and λ-carrageenan, have been reported here. The ability of these materials to uptake the antibiotic ciprofloxacin from aqueous solutions was investigated in several operational conditions. The ACs prepared from κ- and λ-carrageenan have shown a greater adsorption capacity towards ciprofloxacin, when compared to most of the carbonaceous sorbents previously reported, which place these sorbents among the most efficient for this emergent pollutant in the tested conditions. Moreover, the adsorption was very fast, achieving the equilibrium conditions in approximately 5 min. Such good adsorptive performance could be attributed to a combined effect of high specific surface area (above 2300 m^2^/g), high microporosity (near 1 cm^3^/g) and high carbon content. The kinetics and equilibrium modelling analysis indicate that the chemisorption of CIP molecules onto ACs surface is the rate limiting step and that equilibrium sorption is well described by Sips isotherm. These findings demonstrate the potential of ACs prepared from carrageenan polymers as adsorbents for removal of antibiotic pollutants from water. With the aim of evaluating these sorbents in realistic conditions, subsequent studies are planned using real water samples contaminated with ciprofloxacin. The study of the regeneration and reuse of the ACs should also be considered due to economic reasons. Based on previous findings, several treatments could be tested in the regeneration of ACs loaded with ciprofloxacin [13,67,68,69,70,71,72]. An alternative fate of spent sorbents that takes advantage of the potential of ciprofloxacin as a corrosion inhibitor [73] is their use as a resource in the preparation of other materials, following a circular economy approach.

## Figures and Tables

**Table 1:** Morphological and compositional properties of the hydrochars and activated carbons.

Sample	*D_SEM_* (µm/nm) ^a^	C (wt%) ^b^	H (wt%) ^b^	S (wt%) ^b^	O (wt%) ^b^
HC-κ	3.98 ± 1.63 µm	70.734	4.453	u.l.d. ^c^	24.813
HC-ι	2.97 ± 1.18 µm	71.214	4.479	0.356	23.951
HC-λ	4.92 ± 0.48 µm	67.141	4.112	u.l.d ^c^	28.747
AC-κ	45.9 ± 22.8 nm	89.592	0.592	u.l.d ^c^	9.816
AC-ι	157.3 ± 51.5 nm	73.315	3.266	u.l.d ^c^	23.419
AC-λ	54.1 ± 14.3 nm	78.879	0.418	u.l.d ^c^	20.703

^a^ Particle diameter measured using SEM. ^b^ Carbon, hydrogen and sulfur content measured by elemental microanalysis. Oxygen content calculated by difference ^c^ under the limit of instrumental detection.

**Table 2:** Textural properties of the hydrochars and activated carbons.

Sample	*S_BET_* (m^2^/g)	*V_T_*(cm^3^/g)	*V_micro_* (cm^3^/g)	*d_P_* (nm)
HC-κ	4.88	0.0070	--	5.4
HC-ι	30.44	0.0245	--	3.8
HC-λ	11.89	0.0107	--	5.2
AC-κ	2345.6	1.336	0.836	2.3
AC-ι	2804.9	1.229	1.087	1.8
AC-λ	2515.8	1.164	0.968	1.9

**Table 3:** Equilibrium model parameters and goodness of fittings.

Isotherm	Sample	Model Parameters			Goodness of Fit	
**Langmuir**		***q_L_* (mg·g^−1^)**	***K_L_* (L·mg^−1^)**		***R^2^***	**χ^2^**
	AC-κ	410.1	3.616		0.9494	40.06
	AC-ι	327.5	3.907		0.9808	7.412
	AC-λ	435.6	2.941		0.8528	131.1
**Freundlich**		***K_F_* (mg^(1−1/n)^·L^(1/n)^·g^−1^)**	***n***		***R^2^***	**χ^2^**
	AC-κ	245.6	4.991		0.8943	66.59
	AC-ι	215.0	7.314		0.8431	79.09
	AC-λ	245.6	4.912		0.9315	45.27
**Sips**		***N_T_*** **(mg·g^−1^)**	***a* (L·mg^−1^)**	***m***	***R^2^***	**χ^2^**
	AC-κ	460.9	1.732	0.6913	0.9650	28.77
	AC-ι	327.4	3.907	1.0	0.9808	7.412
	AC-λ	1562.5	0.252	0.1909	0.9315	46.07

*q_L_* is the monolayer adsorption capacity. *K_L_* is the Langmuir isotherm constant. *K_F_* is the Freundlich constant. *n* is a dimensionless constant associated with a heterogeneity factor. *N_T_* is the total number of binding sites. *a* is related to the median binding affinity. *m* is the heterogeneous index.

**Table 4:** Kinetic parameters estimated from pseudo 1st order and pseudo 2nd order models and the evaluation of its fittings for an initial CIP concentration of 50 mg/L.

Sample	Pseudo 1st Order			Pseudo 2nd Order		
	*R^2^* (χ^2^)	*k_1_* (min^−1^)	*q_e_* (mg·g^−1^)	*R^2^* (χ^2^)	*k_2_* (g·mg^−1^·min^−1^)	*q_e_* (mg·g^−1^)
AC-κ	0.9999 (5.7 × 10^−3^)	2.677	100.1	0.9999 (4.3 × 10^−3^)	0.1309	100.5
AC-ι	0.9999 (7.2 × 10^−5^)	5.009	100.2	0.9999 (1.6 × 10^−4^)	1.459	100.2
AC-λ	0.9999 (8.6 × 10^−4^)	3.424	100.1	0.9999 (2.6 × 10^−3^)	0.2908	100.2

**Table 5:** Comparison of the maximum CIP adsorption capacity (*q_max_*), time to achieve the equilibrium (*t_e_*) and specific surface area (*S_BET_*) of various carbonaceous adsorbents (T = 25 °C).

Adsorbent	*q_max_* (mg/g)	*S_BET_* (m_2_/g)	*t_e_*	pH	Reference
AC-κ	422	2346	5 min	6	(this work)
AC-ι	330	2805	5 min	6	(this work)
AC-λ	459	2516	5 min	6	(this work)
Powder activated carbon	109	1075	>2 h	6.2	[64]
AC_bamboo_/H_3_PO_4_	613.0	2237	>2 h	6	[24]
AC_peach-stones/_H_3_PO_4_	263.7	1521	>4 h	6.5	[22]
AC_wood_/H_3_PO_4_	231.0	1237	20 h	5	[20]
AC_albizia-seed_/Microwave	131	1824	>2 h	9	[65]
Multiwalled Carbon Nanotubes	206	382	>1 h	4	[21]
AC_palm leaflets_	116.3	24.4	48 h	6	[13]
Magnetic Carbon Nanocomposite	98.28	26.5	24 h	7	[23]

## Supplementary Materials

The following are available online at http://www.mdpi.com/2079-4991/8/12/1004/s1, Figure S1: Powder XRD pattern of HC and AC materials, Table S1: Selected infrared bands (cm^−1^) for the materials and respective assignments, Figure S2: Speciation of CIP, Table S2: Isotherm models and parameters, Table S3: Kinetic models and parameters. Figure S3: Time profile of CIP adsorption capacity over 24h and corresponding kinetic model fitting.

## References

[1] Sousa J.C.G., Ribeiro A.R., Barbosa M.O., Pereira M.F.R., Silva A.M.T. (2018). A review on environmental monitoring of water organic pollutants identified by EU guidelines. J. Hazard. Mater..

[2] Radović T., Grujić S., Petković A., Dimkić M., Laušević M. (2015). Determination of pharmaceuticals and pesticides in river sediments and corresponding surface and ground water in the Danube River and tributaries in Serbia. Environ. Monit. Assess..

[3] Gavrilescu M., Demnerová K., Aamand J., Agathos S., Fava F. (2015). Emerging pollutants in the environment: Present and future challenges in biomonitoring, ecological risks and bioremediation. N. Biotechnol..

[4] Windsor F.M., Ormerod S.J., Tyler C.R. (2018). Endocrine disruption in aquatic systems: up-scaling research to address ecological consequences. Biol. Rev..

[5] Deblonde T., Cossu-Leguille C., Hartemann P. (2011). Emerging pollutants in wastewater: A review of the literature. Int. J. Hyg. Environ. Health.

[6] Paíga P., Correia M., Fernandes M.J., Silva A., Carvalho M., Vieira J., Jorge S., Silva J.G., Freire C., Delerue-Matos C. (2019). Assessment of 83 pharmaceuticals in WWTP influent and effluent samples by UHPLC-MS/MS: Hourly variation. Sci. Total Environ..

[7] Khattab F., Salem H., Riad S., Elbalkiny H. (2014). Determination of fluoroquinolone antibiotics in industrial wastewater by high-pressure liquid chromatography and thin-layer chromatography-densitometric methods. J. Planar Chromatogr. Mod. TLC.

[8] Pereira A.M.P.T., Silva L.J.G., Lino C.M., Meisel L.M., Pena A. (2016). Assessing environmental risk of pharmaceuticals in Portugal: An approach for the selection of the Portuguese monitoring stations in line with Directive 2013/39/EU. Chemosphere.

[9] Ahmadzadeh S., Asadipour A., Pournamdari M., Behnam B., Reza Rahimi H., Dolatabadi M. (2017). Removal of ciprofloxacin from hospital wastewater using electrocoagulation technique by aluminum electrode: Optimization and modelling through response surface methodology. Process Saf. Environ. Prot..

[10] Rehman M.S.U., Rashid N., Ashfaq M., Saif A., Ahmad N., Han J.I. (2015). Global risk of pharmaceutical contamination from highly populated developing countries. Chemosphere.

[11] Riaz L., Mahmood T., Khalid A., Rashid A., Ahmed Siddique M.B., Kamal A., Coyne M.S. (2018). Fluoroquinolones (FQs) in the environment: A review on their abundance, sorption and toxicity in soil. Chemosphere.

[12] Hernández F., Calısto-Ulloa N., Gómez-Fuentes C.M., Gómez J.F., González-Rocha G., Bello-Toledo H., Botero-Coy A.M., Boıx C., Ibáñez M., Montory M. (2018). Occurrence of antibiotics and bacterial resistance in wastewater and sea water from the Antarctic. J. Hazard. Mater..

[13] El-Shafey E.S.I., Al-Lawati H., Al-Sumri A.S. (2012). Ciprofloxacin adsorption from aqueous solution onto chemically prepared carbon from date palm leaflets. J. Environ. Sci..

[14] Krzeminski P., Tomei M.C., Karaolia P., Langenhoff A., Almeida C.M.R., Felis E., Gritten F., Andersen H.R., Fernandes T., Manaia C.M. (2019). Performance of secondary wastewater treatment methods for the removal of contaminants of emerging concern implicated in crop uptake and antibiotic resistance spread: A review. Sci. Total Environ..

[15] Mondal S.K., Saha A.K., Sinha A. (2018). Removal of ciprofloxacin using modified advanced oxidation processes: Kinetics, pathways and process optimization. J. Clean. Prod..

[16] Li W., Guo C., Su B., Xu J. (2012). Photodegradation of four fluoroquinolone compounds by titanium dioxide under simulated solar light irradiation. J. Chem. Technol. Biotechnol..

[17] Stadlmair L.F., Letzel T., Drewes J.E., Grassmann J. (2018). Enzymes in removal of pharmaceuticals from wastewater: A critical review of challenges, applications and screening methods for their selection. Chemosphere.

[18] Yu F., Li Y., Han S., Ma J. (2016). Adsorptive removal of antibiotics from aqueous solution using carbon materials. Chemosphere.

[19] De Andrade J.R., Oliveira M.F., Da Silva M.G.C., Vieira M.G.A. (2018). Adsorption of Pharmaceuticals from Water and Wastewater Using Nonconventional Low-Cost Materials: A Review. Ind. Eng. Chem. Res..

[20] Carabineiro S.A.C., Thavorn-Amornsri T., Pereira M.F.R., Serp P., Figueiredo J.L. (2012). Comparison between activated carbon, carbon xerogel and carbon nanotubes for the adsorption of the antibiotic ciprofloxacin. Catal. Today.

[21] Yu F., Sun S., Han S., Zheng J., Ma J. (2016). Adsorption removal of ciprofloxacin by multi-walled carbon nanotubes with different oxygen contents from aqueous solutions. Chem. Eng. J..

[22] Álvarez-Torrellas S., Peres J.A., Gil-Álvarez V., Ovejero G., García J. (2017). Effective adsorption of non-biodegradable pharmaceuticals from hospital wastewater with different carbon materials. Chem. Eng. J..

[23] Shi S., Fan Y., Huang Y. (2013). Facile low temperature hydrothermal synthesis of magnetic mesoporous carbon nanocomposite for adsorption removal of ciprofloxacin antibiotics. Ind. Eng. Chem. Res..

[24] Wang Y.X., Ngo H.H., Guo W.S. (2015). Preparation of a specific bamboo based activated carbon and its application for ciprofloxacin removal. Sci. Total Environ..

[25] Zhang B., Han X., Gu P., Fang S., Bai J. (2017). Response surface methodology approach for optimization of ciprofloxacin adsorption using activated carbon derived from the residue of desilicated rice husk. J. Mol. Liq..

[26] Mansour F., Al-Hindi M., Yahfoufi R., Ayoub G.M., Ahmad M.N. (2017). The use of activated carbon for the removal of pharmaceuticals from aqueous solutions: a review. Rev. Environ. Sci. Biotechnol..

[27] Correa C.R., Kruse A. (2018). Biobased functional carbon materials: Production, characterization, and applications-A review. Materials.

[28] Yang Y., Cui J., Zheng M., Hu C., Tan S., Xiao Y., Yang Q., Liu Y. (2012). One-step synthesis of amino-functionalized fluorescent carbon nanoparticles by hydrothermal carbonization of chitosan. Chem. Commun..

[29] Marrakchi F., Ahmed M.J., Khanday W.A., Asif M., Hameed B.H. (2017). Mesoporous-activated carbon prepared from chitosan flakes via single-step sodium hydroxide activation for the adsorption of methylene blue. Int. J. Biol. Macromol..

[30] Guan J., Li L., Mao S., Venkatesan J., Anil S., Kim S.-K.B.T.-S.P. (2017). Chapter 15—Applications of Carrageenan in Advanced Drug Delivery.

[31] Stephen A.M., Phillips G.O., Williams P.A. (2006). Food Polysaccharides and Their Applications.

[32] Nanaki S.G., Kyzas G.Z., Tzereme A., Papageorgiou M., Kostoglou M., Bikiaris D.N., Lambropoulou D.A. (2015). Synthesis and characterization of modified carrageenan microparticles for the removal of pharmaceuticals from aqueous solutions. Colloids Surf. B Biointerfaces.

[33] Soares S.F., Simões T.R., António M., Trindade T., Daniel-da-Silva A.L. (2016). Hybrid nanoadsorbents for the magnetically assisted removal of metoprolol from water. Chem. Eng. J..

[34] Fernandes T., Soares S., Trindade T., Daniel-da-Silva A. (2017). Magnetic Hybrid Nanosorbents for the Uptake of Paraquat from Water. Nanomaterials.

[35] Papageorgiou M., Nanaki S.G., Kyzas G.Z., Koulouktsi C., Bikiaris D.N., Lambropoulou D.A. (2017). Novel isocyanate-modified carrageenan polymer materials: Preparation, characterization and application adsorbent materials of pharmaceuticals. Polymers.

[36] Raymundo-Piñero E., Cadek M., Béguin F. (2009). Tuning carbon materials for supercapacitors by direct pyrolysis of seaweeds. Adv. Funct. Mater..

[37] Fan Y., Yang X., Zhu B., Liu P.F., Lu H.T. (2014). Micro-mesoporous carbon spheres derived from carrageenan as electrode material for supercapacitors. J. Power Sources.

[38] ISO (International Organisation for Standardisation) (2010). Determination of the Specific Surface Area of Solids by Gas Adsorption—BET Method (ISO 9277).

[39] Gregg S.J., Sing K.S.W. (1982). Adsorption, Surface Area and Porosity.

[40] Dubinin M.M. (1989). Fundamentals of the theory of adsorption in micropores of carbon adsorbents: Characteristics of their adsorption properties and microporous structures. Carbon N. Y..

[41] Romanos J., Beckner M., Rash T., Firlej L., Kuchta B., Yu P., Suppes G., Wexler C., Pfeifer P. (2012). Nanospace engineering of KOH activated carbon. Nanotechnology.

[42] Chen W., Rakhi R.B., Hedhili M.N., Alshareef H.N. (2014). Shape-controlled porous nanocarbons for high performance supercapacitors. J. Mater. Chem. A.

[43] Zhu Y., Murali S., Stoller M.D., Ganesh K.J., Cai W., Ferreira P.J., Pirkle A., Wallace R.M., Cychosz K.A., Thommes M. (2011). Carbon-Based Supercapacitors Produced by Activation of Graphene. Science.

[44] Rivera-Utrilla J., Sánchez-Polo M., Gómez-Serrano V., Álvarez P.M., Alvim-Ferraz M.C.M., Dias J.M. (2011). Activated carbon modifications to enhance its water treatment applications. An overview. J. Hazard. Mater..

[45] Thommes M., Kaneko K., Neimark A.V., Olivier J.P., Rodriguez-Reinoso F., Rouquerol J., Sing K.S.W. (2015). Physisorption of gases, with special reference to the evaluation of surface area and pore size distribution (IUPAC Technical Report). Pure Appl. Chem..

[46] Dresselhaus M.S., Jorio A., Hofmann M., Dresselhaus G., Saito R. (2010). Perspectives on Carbon Nanotubes and Graphene Raman Spectroscopy. Nano Lett..

[47] Lozano-Castelló D., Lillo-Ródenas M.A., Cazorla-Amorós D., Linares-Solano A. (2001). Preparation of activated carbons from Spanish anthracite - I. Activation by KOH. Carbon N. Y..

[48] Biniak S. (1997). The characterization of activated carbons with oxygen and nitrogen surface groups. Carbon.

[49] Tekin K., Karagöz S., Bektaş S. (2014). A review of hydrothermal biomass processing. Renew. Sustain. Energy Rev..

[50] Bedin K.C., Cazetta A.L., Souza I.P.A.F., Pezoti O., Souza L.S., Souza P.S.C., Yokoyama J.T.C., Almeida V.C. (2018). Porosity enhancement of spherical activated carbon: Influence and optimization of hydrothermal synthesis conditions using response surface methodology. J. Environ. Chem. Eng..

[51] Carmosini N., Lee L.S. (2009). Chemosphere Ciprofloxacin sorption by dissolved organic carbon from reference and bio-waste materials. Chemosphere.

[52] Drakopoulos A.I., Ioannou P.C. (1997). Spectrofluorimetric study of the acid-base equilibria and complexation behavior of the fluoroquinolone antibiotics ofloxacin, norfloxacin, ciprofloxacin and pefloxacin in aqueous solution. Anal. Chim. Acta.

[53] Zhao Q., Zhang S., Zhang X., Lei L., Ma W., Ma C., Song L., Chen J., Pan B., Xing B. (2017). Cation-Pi Interaction: A Key Force for Sorption of Fluoroquinolone Antibiotics on Pyrogenic Carbonaceous Materials. Environ. Sci. Technol..

[54] Ma J., Yang M., Yu F., Zheng J. (2015). Water-enhanced Removal of Ciprofloxacin from Water by Porous Graphene Hydrogel. Sci. Rep..

[55] Langmuir I. (1918). The adsorption of gases on plance surfaces of glass, mica and platnium. J. Am. Chem. Soc..

[56] Freundlich H. (1906). Concerning Adsorption in Solutions. Zeitschrift fur physikalische chemie-stochiometrie und verwandtschaftslehre. Phys. Chem..

[57] Sips R. (1948). On the structure of a catalyst surface. J. Chem. Phys..

[58] Umpleby R.J., Baxter S.C., Chen Y., Shah R.N., Shimizu K.D. (2001). Characterization of Molecularly Imprinted Polymers with the Langmuir-Freundlich Isotherm. Anal. Chem..

[59] Ho Y.S., Porter J.F., McKay G. (2002). Equilibrium isotherm studies for the sorption of divalent metal ions onto peat: Copper, nickel and lead single component systems. Water. Air. Soil Pollut..

[60] McKay G., Allen S.J., McConvey I.F., Walters J.H.R. (1984). External mass transfer and homogeneous solid-phase diffusion effects during the adsorption of dyestuffs. Ind. Eng. Chem. Process Des. Dev..

[61] Grégorio C., Lichtfouse E.D., Wilson L., Morin-Crini N. (2018). Adsorption-Oriented Processes Using Conventional and Non-conventional Adsorbents for Wastewater Treatment. Green Adsorbents for Pollutant Removal.

[62] Lagergren S. (1989). Zur Theorie der Sogenannten Adsorption Gelöster Stoffe, Kungliga Svenska Vetenskapsakademiens. Handlingar.

[63] Ho Y.S., McKay G. (1999). Pseudo-second order model for sorption processes. Process Biochem..

[64] Li X., Chen S., Fan X., Quan X., Tan F., Zhang Y., Gao J. (2015). Adsorption of ciprofloxacin, bisphenol and 2-chlorophenol on electrospun carbon nanofibers: In comparison with powder activated carbon. J. Colloid Interface Sci..

[65] Ahmed M.J., Theydan S.K. (2014). Fluoroquinolones antibiotics adsorption onto microporous activated carbon from lignocellulosic biomass by microwave pyrolysis. J. Taiwan Inst. Chem. Eng..

[66] Mailler R., Gasperi J., Coquet Y., Deshayes S., Zedek S. (2015). Study of a large scale powdered activated carbon pilot: Removals of a wide range of emerging and priority micropollutants from wastewater treatment plant effluents. Water Res..

[67] Snyder S.A., Adham S., Redding A.M., Cannon F.S., Decarolis J., Oppenheimer J., Wert E.C., Yoon Y. (2007). Role of membranes and activated carbon in the removal of endocrine disruptors and pharmaceuticals. Desalination.

[68] Purkait M.K., Maiti A., DasGupta S., De S. (2007). Removal of congo red using activated carbon and its regeneration. J. Hazard. Mater..

[69] Matatov-Meytal Y.I., Sheintuch M. (1997). Abatement of Pollutants by Adsorption and Oxidative Catalytic Regeneration. Ind. Eng. Chem. Res..

[70] Kim J.H., Ryu Y.K., Haam S., Lee C.H., Kim W.S. (2001). Adsorption and steam regeneration of n-hexane, MEK, and toluene on activated carbon fiber. Sep. Sci. Technol..

[71] Hassanzadeh S., Aminlashgari N., Hakkarainen M. (2014). Microwave-assisted recycling of waste paper to green platform chemicals and carbon nanospheres. ACS Sustain. Chem. Eng..

[72] Fang C.S., Lai P.M.C. (1996). Microwave Regeneration of Spent Powder Activated Carbon. Chem. Eng. Commun..

[73] Eddy N.O., Stoyanov S.R., Ebenso E.E. (2010). Fluoroquinolones as corrosion inhibitors for mild steel in acidic medium; Experimental and theoretical studies. Int. J. Electrochem. Sci..

