# Supplementary material for: Porous Carrageenan-Derived Carbons for Efficient Ciprofloxacin Removal from Water
Source: Nanomaterials (Basel). 2018 Dec 4;8(12):1004. doi: 10.3390/nano8121004 (PMC6316754; doi:10.3390/nano8121004)
Supplement: Supplementary file 1 [file nanomaterials-08-01004-s001.pdf]

nanomaterials

MDPI

Supporting Information

Porous carrageenan-derived carbons for efficient ciprofloxacin removal from water

João Nogueira [1], Maria António [1], Sergey M. Mikhalev [2], Sara Fateixa [1], Tito Trindade [1] and Ana L. Daniel-da-Silva [1,*]

[1] CICECO-Aveiro Institute of Materials, Department of Chemistry, University of Aveiro, 3810-193 Aveiro, Portugal; jh.nogueira@ua.pt (J.N.); maantonio@ua.pt (M.A.); sarafateixa@ua.pt (S.F.); tito@ua.pt (T.T.)

[2] Centre for Mechanical Technology and Automation – Nanotechnology Research Group (TEMA-NRD), Mechanical Engineering Department, Aveiro Institute of Nanotechnology (AIN), University of Aveiro, 3810-193 Aveiro, Portugal; mikhalev@ua.pt

* Correspondence: ana.luisa@ua.pt; Tel.: +351-234-370-368

A. Characterization of carbon materials

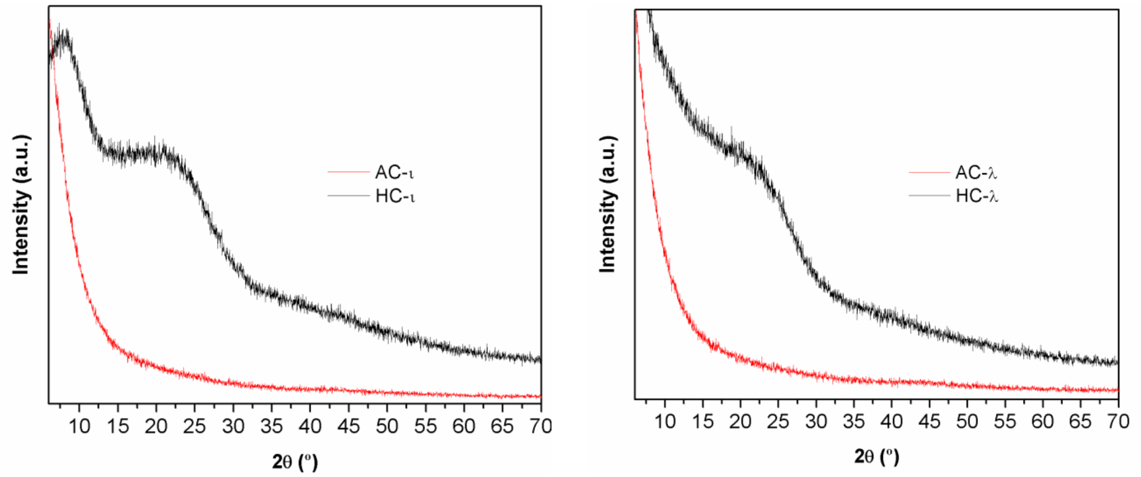

Figure S1. Powder XRD pattern of carrageenan (ι- and λ-type; left and right, respectively) derived carbons before (HC) and after activation (AC).

Table S1. Selected infrared bands (cm^{-1}) for the materials and respective assignments[a].

	Hydrothermal carbons			Activated carbons		
**Assignment**	**HC-κ**	**HC-ι**	**HC-λ**	**AC-κ**	**AC-ι**	**AC-λ**
ν(O-H)	3273 (vs)	3221 (vs)	3315 (vs)	--	--	--
ν(C-H) aliphatic	2926 (s) 2879 (s)	2922 (s) 2879 (s)	2926 (s) 2879 (s)	--	--	--
ν(C=O)	1693 (s)	1695 (s)	1695 (s)	--	--	--
ν(C=C) aromatic	1606 (vs)	1606 (vs)	1606 (vs)	1560-1520 (vs)	1560-1520 (vs)	1560-1520 (m)
δ(C-O)	1290 (vs)	1292 (vs)	1298 (vs)	--	--	--
C-H aromatic	798 (s)	798 (s)	798 (s)	--	--	--

[a] vs – very strong; s – strong; m– medium; ν – stretching vibration; δ – deformation vibration

B. CIP Adsorption Modelling

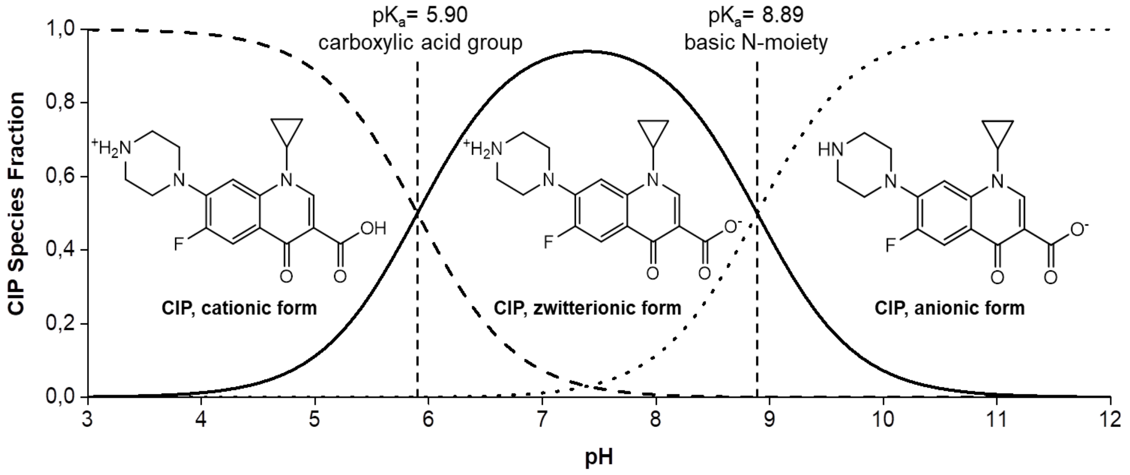

Figure S2. Speciation of CIP [1,2]

Table S2. Isotherm models and parameters [3–6].

Model	Equation (non-linear form)	Parameters
**Langmuir**	$q_e = \frac{q_L K_L C_e}{1 + K_L C_e}$	q_L is the monolayer adsorption capacity (mg.g^{-1}) K_L is the Langmuir isotherm constant (L.mg^{-1}), related to the affinity of binding sites.
**Freundlich**	$q_e = K_F C_e^{\frac{1}{n}}$	K_F is the Freundlich constant (mg$^{(1-1/n)}$.L$^{(1/n)}$.g^{-1}) 1/n is the heterogeneity factor; n is usually between 1 and 10 (dimensionless)
**Sips**	$q_e = \frac{N_T a C_e^m}{1 + a C_e^m}$	N_T is the total number of binding sites (mg.g^{-1}) a is related to the median binding affinity k ($a=k^m$) m is the heterogeneous index (dimensionless, $0<m<1$)

Table S3. Kinetic models and parameters. q_t and q_e (mg. g^{-1}) are the adsorption capacity at time t and equilibrium time, respectively [7,8].

Model	Equation (non-linear form)	Parameters
**Pseudo 1st order**	$q_t = q_e(1 - e^{-k_1 t})$	k_1 - equilibrium rate constant of pseudo 1st order adsorption (min^{-1}).
**Pseudo 2nd order**	$q_t = \frac{k_2 {q_e}^2 t}{1 + k_2 q_e t}$	k_2 - equilibrium rate constant of pseudo 2nd order adsorption (g.mg^{-1}.min^{-1}).

The goodness of the fittings was determined based on the calculation of the correlation coefficient (R^2) (S1) and Chi-square test value (χ^2) (S2), expressed by the following equations respectively:

$$R^2 = 1 - \frac{\sum_{i=1}^{n}(y_i - \hat{y}_i)^2}{\sum_{i=1}^{n}(y_i - \bar{y})^2} \quad \text{(S1)} \qquad \chi^2 = \sum_{i=1}^{n}\frac{(y_i - \hat{y}_i)^2}{\hat{y}_i} \quad \text{(S2)}$$

where y_i and $\hat{y}_i$ are the experimental and model predicted values respectively, $\bar{y}$ is the mean of the experimental data and n is the sample size.

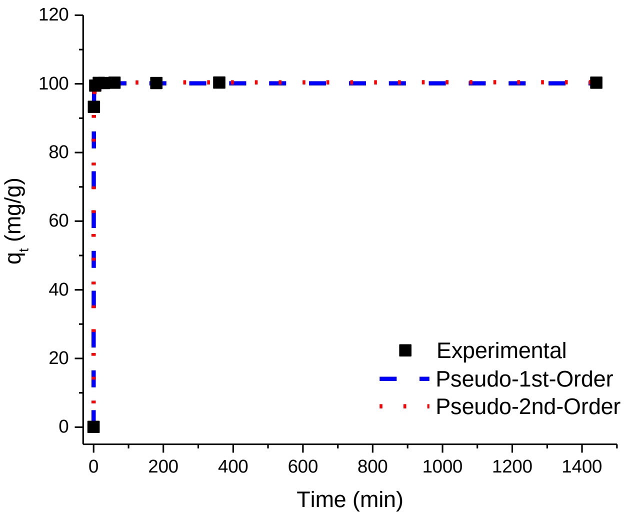

(**a**)

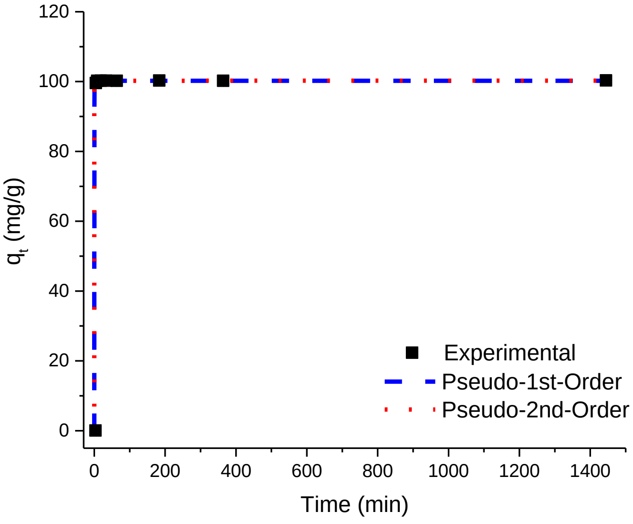

(**b**)

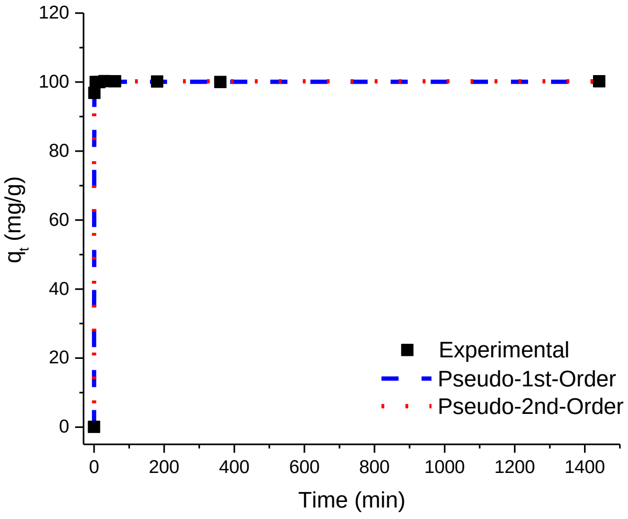

(**c**)

Figure S3. Time profile of CIP adsorption capacity over 24h and corresponding kinetic model fitting using pseudo 1st and pseudo 2nd order equations: AC-κ (a), AC-ι (b), AC-λ (c).

References

1. Carmosini, N.; Lee, L.S. Chemosphere Ciprofloxacin sorption by dissolved organic carbon from reference and bio-waste materials. *Chemosphere* **2009**, *77*, 813–820, doi:10.1016/j.chemosphere.2009.08.003.
2. Drakopoulos, A.I.; Ioannou, P.C. Spectrofluorimetric study of the acid-base equilibria and complexation behavior of the fluoroquinolone antibiotics ofloxacin , norfloxacin , ciprofloxacin and pefloxacin in aqueous solution. *Anal. Chim. Acta* **1997**, *354*, 197–204.
3. Langmuir, I. The adsorption of gases on plance surfaces of glass, mica and platnium. *J. Am. Chem. Soc.* **1918**, *40*, 1361–1403, doi:10.1021/ja02242a004.
4. Freundlich, H. Concerning Adsorption in Solutions. Zeitschrift fur physikalische chemie-stochiometrie und verwandtschaftslehre. *Phys. Chem.* **1906**, *57*, 385–470.
5. Sips, R. On the structure of a catalyst surface. *J. Chem. Phys.* **1948**, *16*, 490–495, doi:10.1007/978-1-4614-5836-4_45.
6. Umpleby, R.J.; Baxter, S.C.; Chen, Y.; Shah, R.N.; Shimizu, K.D. Characterization of Molecularly Imprinted Polymers with the Langmuir-Freundlich Isotherm. *Anal. Chem.* 2001, *73*, 4584–4591.
7. Lagergren, S. Zur Theorie der Sogenannten Adsorption Gelöster Stoffe, Kungliga Svenska Vetenskapsakademiens. *Handlingar* **1989**, *24*, 1–39.
8. Ho, Y.S.; McKay, G. Pseudo-second order model for sorption processes. *Process Biochem.* **1999**, *34*, 451–465, doi:10.1016/S0032-9592(98)00112-5.

© 2018 by the authors. Submitted for possible open access publication under the terms and conditions of the Creative Commons Attribution (CC BY) license (http://creativecommons.org/licenses/by/4.0/).
